# Eating Disorders and Dental Erosion: A Systematic Review

**DOI:** 10.3390/jcm12196161

**Published:** 2023-09-24

**Authors:** Kacper Nijakowski, Jakub Jankowski, Dawid Gruszczyński, Anna Surdacka

**Affiliations:** 1 Department of Conservative Dentistry and Endodontics, Poznan University of Medical Sciences, 60-812 Poznan, Poland; annasurd@ump.edu.pl; 2 Student’s Scientific Group in Department of Conservative Dentistry and Endodontics, Poznan University of Medical Sciences, 60-812 Poznan, Poland; jjankowski41@wp.pl (J.J.); dawid.j.gruszczynski@gmail.com (D.G.)

**Keywords:** eating disorders, anorexia nervosa, bulimia nervosa, dental erosion, tooth erosion

## Abstract

Both eating disorders and dental erosion are increasingly affecting adolescents and young adults. Thus, our systematic review was designed to answer the question: “Is there a relationship between dental erosion and eating disorders?” Following the inclusion and exclusion criteria, 31 studies were included in this systematic review (according to the PRISMA statement guidelines). Based on the meta-analysis, 54.4% of patients with bulimia nervosa and 26.7% with anorexia nervosa experienced tooth erosion. For the whole group of 1699 patients with eating disorders, erosive lesions were observed in 42.1% of patients. Bulimics were more than 10 times more likely to experience dental erosion compared to healthy individuals (OR = 10.383 [95%CI: 4.882–22.086]). Similarly, more than 16 times increased odds of tooth erosion were found in patients with self-induced vomiting (OR = 16.176 [95%CI: 1.438–181.918]). In conclusion, eating disorders are associated with an increased risk of developing erosive lesions, especially in patients with bulimia nervosa.

## 1. Introduction

Eating disorders (EDs) are serious mental and behavioural disorders that may occur alone or in combination with other mood disorders [1]. Due to the global trend towards beauty and fitness, the incidence of these disorders among adolescents and young adults is rapidly increasing [2]. Although EDs are most commonly reported in young women, they can affect people of both genders at any life stage [3,4]. The etiology of these disorders is not fully understood, but it is assumed that it is multifactorial, depending on individual (biological or psychological, including low self-esteem) and environmental (developmental or sociocultural, especially pressures in home, school, etc.) issues [2,5].

The two most common ED forms include anorexia nervosa (AN) and bulimia nervosa (BN) [1,6]. AN is mainly associated with food restriction, leading to significant underweight. Patients with AN are characterised by an impaired vision of their own body shape and extreme fear of gaining weight [7,8]. In turn, BN is more common and is usually associated with normal body weight. Patients with BN demonstrate recurrent uncontrolled binge-eating episodes which are inappropriately compensated by such behaviours as self-induced vomiting, purging or overexercising [9,10].

Due to their pathophysiology, both types affect the person’s general health, as well as the oral health [11]. The first symptoms can sometimes be observed in the oral cavity [12]. In patients with EDs, the most common oral symptoms include gingivitis or periodontitis, mucosal ulcers or erythema, angular cheilitis, xerostomia, as well as dental caries or erosion [13,14,15]. 

Tooth erosion is caused by the supply of acids of non-bacterial origin [16]. Food products with a low pH (e.g., fruits, fresh juices) are most often considered as etiological factors [17]. However, internal factors such as eating disorders (especially those with recurrent vomiting) are also known to cause erosion [18]. In addition, regular physical activity with supplementation of sports drinks strongly increases the risk of dental erosion [19,20,21]. 

It is now assumed that tooth erosion has become a new dental plague in a group of adolescents and young adults [22,23]. Under the influence of acids delivered to the oral cavity, the enamel dissolves, and thus erosive lesions develop [24]. It is particularly important to prevent dental erosion and eating disorders through appropriate education and prophylaxis from an early age [25,26]. The preventive actions include the right home oral hygiene manoeuvres; e.g., the use of toothpastes with fluoride [27]. Fluoridated toothpastes provide a higher degree of remineralisation at a first acid attack [28].

Therefore, our systematic review was designed to answer the question: “Is there a relationship between dental erosion and eating disorders?”.

## 2. Materials and Methods

### 2.1. Search Strategy and Data Extraction

The present systematic review was conducted up to 27 August 2023, according to the Preferred Reporting Items for Systematic Reviews and Meta-Analyses (PRISMA) statement guidelines [29], using the PubMed, Scopus and Web of Science databases. The search queries included:

- for PubMed: (eating disorders OR anorexia OR bulimia) AND ((tooth OR dental) AND erosion)
- for Scopus: TITLE-ABS-KEY((“eating disorders” OR anorexia OR bulimia) AND ((tooth OR dental) AND erosion))
- for Web of Science: TS = ((eating disorders OR anorexia OR bulimia) AND ((tooth OR dental) AND erosion)).

Records were screened according to the title, abstract and full text by two independent investigators. The studies included in this review matched all the predefined criteria according to PI(E)COS (“Population”, “Intervention”/”Exposure”, “Comparison”, “Outcomes” and “Study design”), as reported in Table 1. A detailed search flowchart is presented in the Results section. The study protocol was registered in the international prospective register of systematic reviews, PROSPERO (CRD42023458225).

The results of the meta-analysis were presented in forest plots using the MedCalc Statistical Software, version 19.5.3 (MedCalc Software Ltd., Ostend, Belgium). The meta-analysis was performed using the subgroups of anorexia nervosa and bulimia nervosa, as well as mixed eating disorders (additionally with and without self-induced vomiting). The pooled proportions and odds ratios for the prevalence of dental erosion were calculated.

### 2.2. Quality Assessment and Critical Appraisal for the Systematic Review of Included Studies

The risk of bias in each individual study was assessed according to the “Study Quality Assessment Tool” issued by the National Heart, Lung and Blood Institute within the National Institute of Health [30]. These questionnaires were answered simultaneously by two independent investigators, and any disagreements were resolved through discussion between them. The summarised quality assessment is reported in the Results section. 

The level of evidence was assessed using the classification of the Oxford Centre for Evidence-Based Medicine levels for diagnosis [31].

## 3. Results

### 3.1. Search Strategy

**Figure 1:**
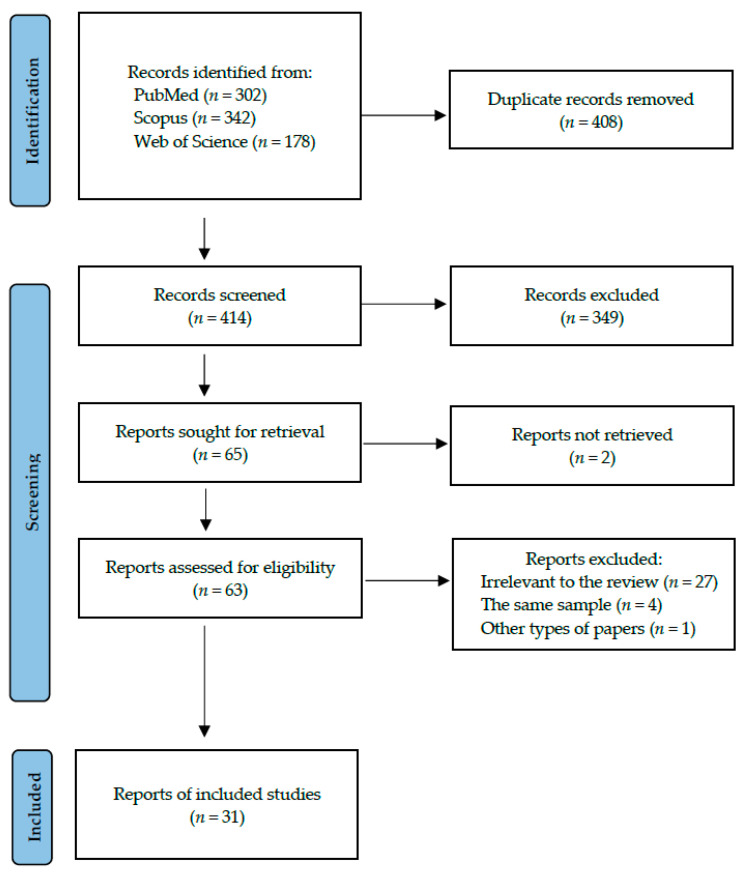
PRISMA flow diagram presenting search strategy.

Following the search criteria, our systematic review included 31 studies, demonstrating data collected in 17 different countries from a total of 1699 participants with EDs (mainly BN and AN). Figure 1 reports the detailed selection strategy of the records. The inclusion and exclusion criteria are presented in the Materials and Methods section.

### 3.2. Characteristics of Included Studies

As shown in Table 2, we collected data about the characteristics of each eligible study, such as the year of publication and study setting, age and gender distribution of study and control groups, diagnoses of EDs and clinical criteria for dental erosion. The vast majority of the participants were young women. Most studies had a case-control design. In terms of the types of EDs, a significant proportion were bulimics, followed by anorectics; however, some studies did not separate these subgroups to assess the oral health. Due to the very broad timeframe, the diagnostic criteria for dental erosion were very diverse, although in the latest studies, the standard was already BEWE (Basic Erosive Wear Examination).

### 3.3. Quality Assessment of Included Studies

**Figure 2:**
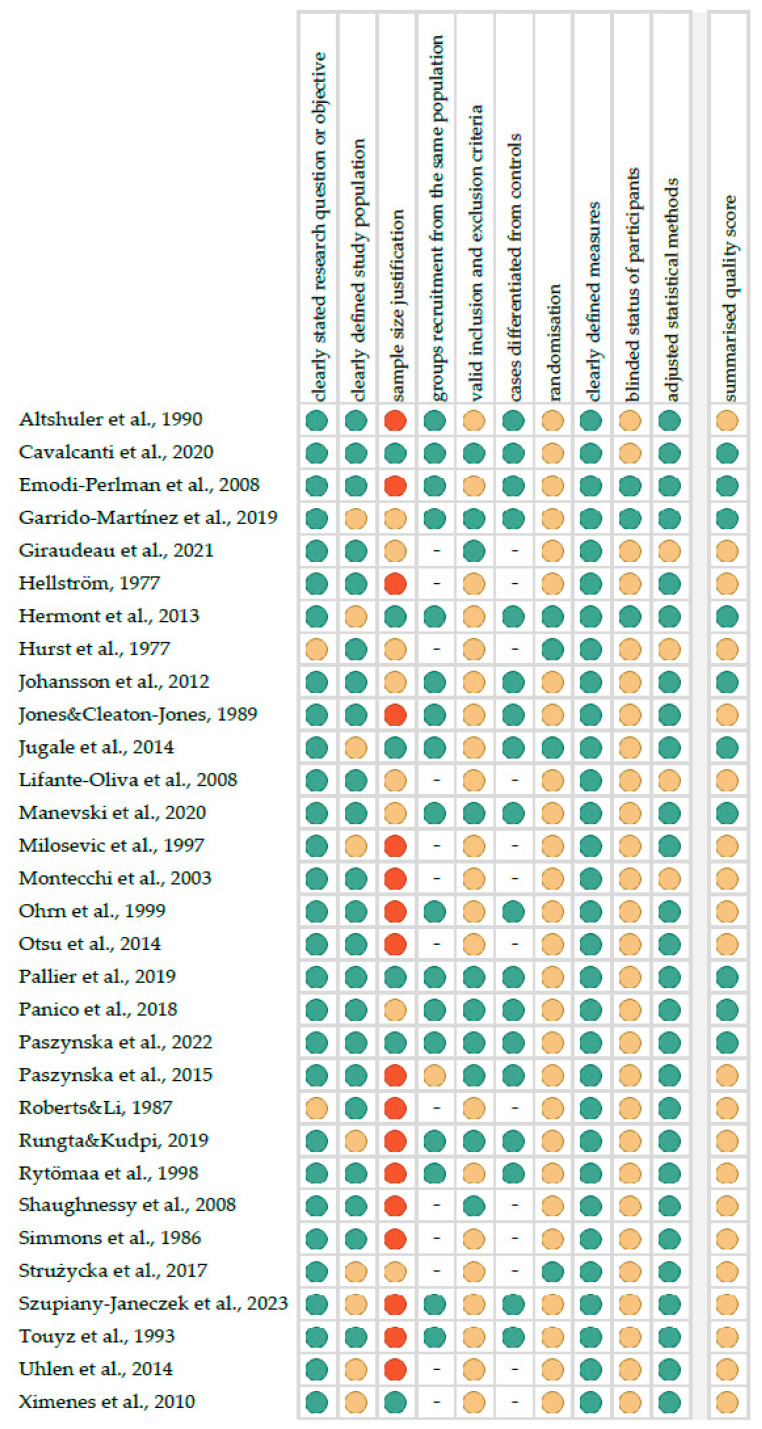
Quality assessment, including the main potential risk of bias (risk level: green—low, yellow—unspecified, red—high; quality score: green—good, yellow—intermediate, red—poor) [32,33,34,35,36,37,38,39,40,41,42,43,44,45,46,47,48,49,50,51,52,53,54,55,56,57,58,59,60,61,62].

Figure 2 reports the summarised quality assessment, according to the “Study Quality Assessment Tool” issued by the National Heart, Lung and Blood Institute within the National Institute of Health [30]. The most frequently encountered risks of bias were the absence of data regarding blinding (twenty-eight studies), randomisation (twenty-seven studies), sample size justification (twenty-five studies), as well as valid inclusion and exclusion criteria (twenty-one studies). The critical appraisal was summarised by adding up the points for each criterion of potential risk (points: 1—low, 0.5—unspecified, 0—high). Ten studies (32.3%) were classified as being of “good” quality (≥80% total score) and twenty-one (67.7%) were classified as “intermediate” (≥60% total score).

All of the included studies had the third or fourth level of evidence (case-control or cross-sectional studies), according to the five-graded scale in the classification of the Oxford Centre for Evidence-Based Medicine levels for diagnosis [31].

### 3.4. Results of Meta-Analysis

**Figure 3:**
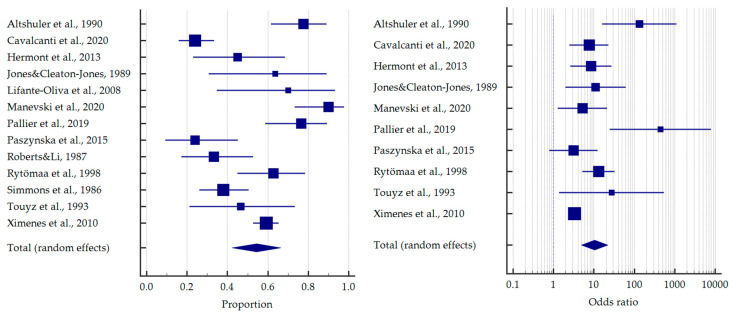
Forest plots presenting the summarised prevalence and odds ratio of dental erosion among patients with bulimia nervosa [32,33,38,41,43,44,49,52,53,55,57,60,62].

As previously mentioned, most of the studies concerned bulimics. Based on the meta-analysis, it was found that more than half of the patients with BN demonstrated tooth erosion—54.4% [95%CI: 42.3–66.3]. Compared to healthy subjects, bulimics were more than 10 times more likely to experience dental erosion—OR = 10.383 [95%CI: 4.882–22.086] (Figure 3).

**Figure 4:**
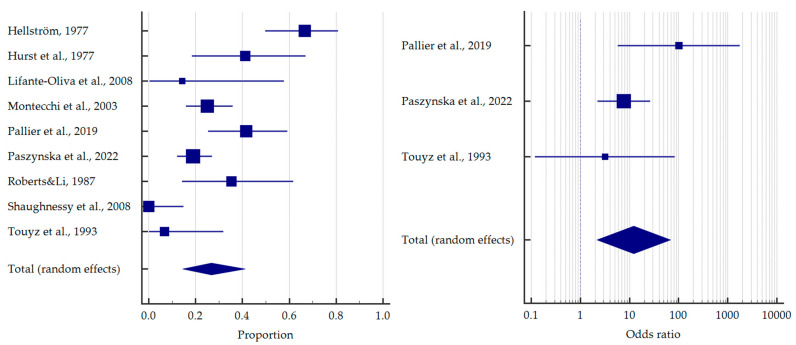
Forest plots presenting the summarised prevalence and odds ratio of dental erosion among patients with anorexia nervosa [37,39,43,46,49,51,53,56,60].

Analogously, more than 1/4 of the anorectic patients had erosive lesions—26.7% [95%CI: 14.5–41.1]. Only three studies compared the frequencies in anorexic and healthy individuals, similarly giving a ten times higher odds of tooth erosion—OR = 12.202 [95%CI: 2.179–68.334] (Figure 4).

**Figure 5:**
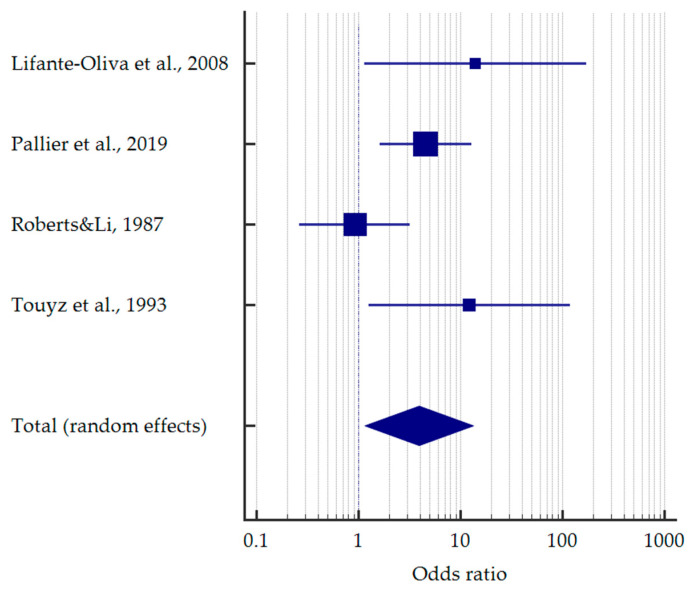
Forest plot presenting the odds ratio of dental erosion among patients with bulimia nervosa vs. anorexia nervosa [43,49,53,60].

However, when comparing bulimics with anorectics, the former had an almost four times higher chance of erosive lesions—OR = 3.926 [95%CI: 1.153–13.365] (Figure 5).

**Figure 6:**
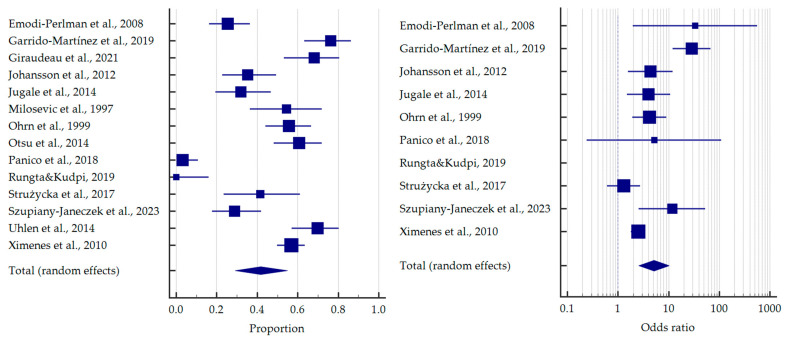
Forest plots presenting the summarised prevalence and odds ratio of dental erosion among other patients with eating disorders (without divided diagnoses) [34,35,36,40,42,45,47,48,50,54,58,59,61,62].

Some of the studies did not separate subgroups of EDs for oral health outcomes. For these studies, the pooled proportion was 41.7% [95%CI: 29.1–54.9] and OR = 5.132 [95%CI: 2.571–10.246] relative to the healthy subjects (Figure 6).

**Figure 7:**
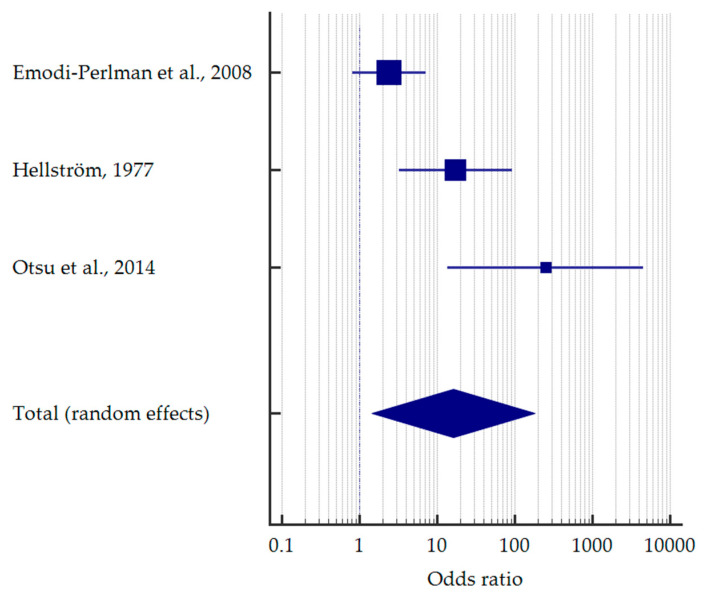
Forest plot presenting the odds ratio of dental erosion among patients with self-induced vomiting [34,37,48].

Interestingly, only three studies singled out self-induced vomiting. Patients with vomiting had more than 16 times higher odds to manifest erosive lesions—OR = 16.176 [95%CI: 1.438–181.918] (Figure 7).

**Figure 8:**
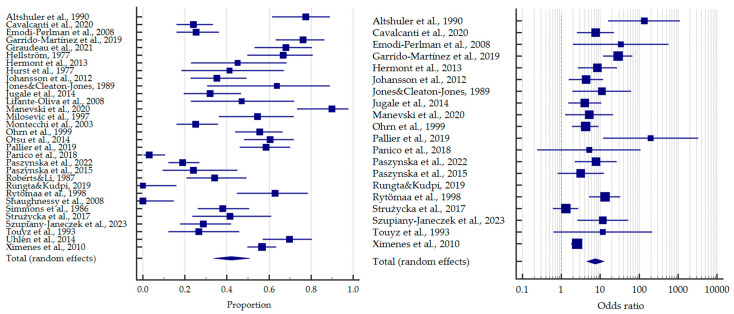
Forest plots presenting the summarised prevalence and odds ratio of dental erosion among all patients with eating disorders [32,33,34,35,36,37,38,39,40,41,42,43,44,45,46,47,48,49,50,51,52,53,54,55,56,57,58,59,60,61,62].

Considering all of the included studies, the incidence of tooth erosion in patients with EDs was 42.1% [95%CI: 33.6–50.8]. In turn, the odds ratio of dental erosion for the whole group relative to the control subjects was 7.480 [95%CI: 4.456–12.556] (Figure 8).

## 4. Discussion

### 4.1. Dental Erosion in Anorexia Nervosa

Many of the included studies considering the impact of AN on the presence of erosive lesions described patients in addiction and psychiatry hospital units, especially young women as AN is a psychosomatic disease mainly affecting this group.

In the last century, many researchers have tried to expand the knowledge regarding oral complications in AN. One of them was Hellström [37], who described 39 patients (38 females and 1 male) aged 14 to 42 years, with AN for periods ranging between 1 and 20 years. These patients were divided into two main groups: the first with vomiting patients and the second with non-vomiting patients. In the group with vomiting patients, severe lingual-occlusal erosion (perimolysis) was nearly always present, but the lingual type did not occur in the non-vomiting cases. Also, the buccal type of erosion was rare in the non-vomiting patients, but it was common in a moderate form in those with vomiting. It was pointed out that this may also be due to the longer history of vomiting with a high consumption of acid beverages. As most of the patients, especially those vomiting, showed different grades of dehydration, to relieve this, they consumed acid drops, juices and lemonade, most often at night.

Other researchers, namely Roberts and Li [53], observed that 35% of the patients with AN in their study showed palatal erosion of the maxillary anterior teeth, which was the result of frequent vomiting. These observations coincide with the study by Lifante-Oliva et al. [43], where, among young female patients with AN, erosive lesions were most often found on the palatal surfaces of the maxillary anterior teeth because these teeth were most often exposed to acidic fluids like vomit. In addition, the authors note that the development of dental erosion may be part of a cumulative process influenced by the frequency and duration of acid exposure, oral hygiene and individual vulnerability. Other factors influencing the presence, extent and advancement of erosion are the patient’s age and AN duration. Therefore, prolonged exposure to the destructive effects of harmful conditions related to AN (vomiting, xerostomia) may significantly deepen or create completely new erosive lesions.

The study by Shaughnessy et al. [56] found no evidence of dental erosion, which was defined as a detectable change in the smooth enamel surface without any indication of dental caries, in any of the young women with a mean AN duration of 2.5 months. However, 26% of the participants reported a history of binge-eating or purging activity. Undoubtedly, the patients’ age and the duration of their AN and vomiting—which is often related to their age—impact the extent and advancement of dental erosion in patients with EDs.

In the same year as Hellström [37], Hurst et al. [39] conducted a study on dental issues, such as erosion, in patients with AN. The study identified three factors that may cause dental problems in these patients: frequent and prolonged vomiting, an unnatural diet and the effects of wasting and dehydration during starvation. The patients were categorised into three groups—vomiters, regurgitators and non-vomiters—based on their vomiting and regurgitation history over the past three years. Like Hellström [37], the researchers noted that dental erosion was significantly more common in the vomiting and regurgitating population than in the non-vomiters. The maxillary incisors, canines and premolars showed dental erosion of the palatal surfaces, resulting in the tooth crown “shelling out”. Despite the majority of the subjects consuming large amounts of fruit regularly, with a preference for citrus fruits, only two subjects displayed significant labial enamel loss due to tooth erosion. However, the researchers point out the low pH of such fruits (3–5) and the possible enamel decalcification after long-term consumption of these foods. The third factor investigated was the intraoral effects of wasting and dehydration of patients during starvation. These authors found that saliva secretion in people with AN is reduced due to starvation and the associated weight-reducing manoeuvres, which lead to the wasting and dehydration of the body. Reducing salivary flow, followed by the acid-buffering capacity of saliva, increases the risk of tooth decay in such patients, and also makes the teeth more susceptible to acid attack and erosion.

Touyz et al. [60] concluded that patients with EDs did not have lower decay incidence or salivary flow compared to the healthy subjects, but did have significantly more acidic saliva. The difference between the study groups was also visible in the frequency of dental erosion. Out of all the surfaces examined, bulimics had 6.1% of surfaces affected and anorexics had 1.0%. In this study, it was found that both anorexics and bulimics had a significantly higher number of surfaces with erosion compared to the control subjects who had no eroded surfaces. 

A significant impact of vomiting on dental erosion was illustrated by Montecchi et al. [46]. They found erosive lesions in 20 of the 80 non-adult patients with AN, especially those who demonstrated vomiting several times daily. Those patients who declared daily vomiting, in addition to tooth erosion, also had carious lesions and xerostomia. Xerostomia in such cases may result from many causes, such as taking drugs that reduce salivation, which can be used in the treatment of AN, or dehydration, which is undoubtedly present after vomiting several times a day.

At present, one of the most frequently used scales by researchers to assess the erosive wear of teeth is the BEWE scoring system [16], which records the most severely affected surface in a sextant. The criteria for the grading are as follows: (0) no defect; (1) initial loss of surface texture; (2) hard tissue loss <50% of the surface area; (3) hard tissue loss ≥50% of the surface area. In a BEWE score of 2 or 3, dentine is often involved. 

Paszynska et al. [51] used this scale to assess dental erosion in 220 female children (117 with AN and 113 controls). They observed a significant difference in the prevalence and severity of dental erosion between these groups. The patients had a BEWE score ≤ 2, which was discovered in 18.9% of cases, while only 2.9% of control subjects had the same score. However, no higher score ≥ 3 was identified in the control group. Erosive lesions were found more often in the patients with AN than in the controls (18.8% vs. 2.9%). In all of the healthy children, the BEWE index was ≤2, while the largest number of AN patients had BEWE 3–8, and the highest result in the AN patients was BEWE 9–13. Also, the researchers divided the AN group into subgroups: those with and without purging episodes. The anorectics without purging had significantly lower BEWE scores. Other researchers [37,46] made the same observations that patients who vomited had a greater severity of dental erosion than those who did not vomit.

Similarly, Pallier et al. [49] observed more AN patients with BEWE ≤ 2 (58.3%) compared to the controls. Overall, 16.7% of the patients with AN had BEWE ≥ 14. However, the authors noticed that the control group included females responding online for a free dental examination. They may be more dentally aware than females in the general population, which is why the BEWE score may be so low. In addition to AN patients, these researchers also examined BN patients, where a BEWE ≤ 2 was assessed in 23.5% and BEWE ≥ 14 in 20.6%. The patients with BN had significantly higher BEWE scores (>2) compared to the patients with AN (76.5% vs. 41.7%).

### 4.2. Dental Erosion in Bulimia Nervosa

The primary cause of dental erosion in bulimic patients is often self-induced vomiting. This results in erosive lesions on the palatal surfaces of the anterior teeth that come into contact with vomit and gastric acid. As a result, many bulimics have severe damage on these surfaces. 

Simmons et al. [57] described the occurrence of dental erosion in patients with BN who self-induced vomiting at least three times a week. Their mean age was 26, and the mean disease duration was 7 years. Overall, 37.9% had significant enamel erosion, and the duration of vomiting was significantly associated with erosion. The erosion prevalence was significantly lower in those who reported vomiting for 4 years or less (24.3%) compared to those who reported vomiting for more than 4 years (55.2%).

The topic of teeth brushing immediately after every vomiting episode was also taken up in this study due to its possible impact on the progression of tooth erosion. Studies have shown that brushing one’s teeth right after vomiting may not prevent erosion but could worsen it. It was found that 50% of patients who brushed their teeth immediately after vomiting had signs of erosion, indicating that brushing may promote enamel loss soon after the acid has already affected the teeth. According to the study by Otsu et al. [48], teeth brushing immediately after vomiting is not recommended. This aligns with prior research indicating that brushing after consuming acidic foods or vomiting can result in acid erosion of tooth surfaces. Additionally, brushing too soon after vomiting may strip away the tooth’s outer layer, revealing the decalcified layer that requires remineralisation over time. Moreover, researchers note that to prevent dental erosion from worsening after vomiting, it is important to thoroughly rinse the mouth with water or other liquids to neutralise any acid present in the oral cavity. 

Previously, such a solution was proposed by Rytömaa et al. [55]. They concluded that such action may have a positive effect in reducing tooth erosion in patients who vomit (including bulimics). Thus, bulimics can prevent tooth damage by quickly neutralizing their stomach contents with water and antacids after vomiting. This is easily achievable because they typically plan their binge eating and vomiting ahead of time.

Erosive lesions resulting from BN often have their characteristic location (as in the case of AN); this was described in the study by Altshuler et al. [32]. The presence of erosive lesions indicates a condition that has persisted for at least six months. They noted an erosion diagnosis when a tooth surface loses enamel, which exposes dentine and/or causes changes to the tooth’s shape. In individuals with BN, 78% showed an average of 7.6 tooth surfaces that were eroded. There was a significant difference compared to the healthy subjects, where the mean was only 0.2 tooth surfaces. The most commonly affected area was the palatal surfaces of the anterior teeth in the maxilla. The other affected areas included the palatal and occlusal surfaces of the posterior teeth in the maxilla, as well as the lingual surfaces of the anterior teeth in the mandible. Only one individual in the control group showed signs of erosion. 

Moreover, Jones and Cleaton-Jones [41] conducted a case-control study on female patients who visited a private dental office. They observed a high prevalence of erosive lesions on the buccal surfaces of the maxillary canines and first premolars, as well as on the palatal surfaces of the same teeth, but a low prevalence on the buccal surfaces of the maxillary incisors. These findings strongly suggest the presence of BN. The study also found that over 50% of the bulimic subjects had erosive defects on these teeth, with over 70% showing lesions on the labial surfaces of the central incisor and canine. Additionally, more than 40% of these patients displayed some degree of dental erosion on the lingual surfaces of the mandibular incisors. It is important to mention that, in this study, erosions were defined as “dished out” areas of enamel or enamel and dentin on the buccal or lingual surface. In addition to the location, the researchers noted the erosion depth and showed that 69% of the bulimics had tooth erosion of any stage, while only 7% of the control group had it. The control group had only enamel erosions, while the bulimic group had erosive lesions at all depths.

A more recent study by Manevski et al. [44] confirmed and summarised the speculations about the location of erosion on tooth surfaces in patients with BN. In total, 83.4% of the patients were students, which corresponds to the fact that BN is most commonly present in the university population [63]. The analysis of the tooth surfaces, based on BEWE, revealed a significant difference between the groups. The majority of the lesions in the bulimic group (43.9%) were located on the palatal/lingual surfaces, while in the control group, the lesions were predominantly found on the labial/buccal surfaces (44%). Erosive lesions on oral surfaces are linked to longer bulimia duration and more frequent vomiting, indicating repeated purging as a crucial etiological factor for dental erosion occurrence in bulimics.

A more frequent occurrence of dental erosion in bulimics was also presented in the study by Paszynska et al. [52]. The results revealed significant differences in tooth wear (TWI) between the bulimic group (24%) and the control group (9%). Only patients with BN showed a total loss of enamel and dentin (over 50% of its thickness) with a TWI score of 3. Also, in the patients with BN, the unstimulated saliva pH was significantly lower than in the healthy controls. Moreover, according to these authors, medications from the selective serotonin reuptake inhibitors, such as fluoxetine, have been proven to be effective in treating bulimic patients without causing salivary alterations.

Comparisons of AN and BN can be seen in other studies by various researchers included in our meta-analysis. As mentioned earlier, in the study by Roberts and Li [53], the AN patients showed palatal erosion of the maxillary anterior teeth. The patients with BN showed erosive lesions in the same place. However, Lifante-Oliva et al. [43] found more patients with BN who had dental erosion than patients with AN. The change in saliva secretion in these patients was also noted, which may be the reason for the increase in erosive lesions. Half of the bulimics presented decreased unstimulated saliva secretion, and 30% of them also showed a reduction in stimulated saliva secretion. In contrast, the anorectics did not demonstrate similar changes. Acidic salivary pH occurred in 20% of the BN patients and 14% of the AN patients. Roberts and Li [53] also revealed that all of the patients diagnosed with BN had a habit of frequent vomiting to control their weight. In contrast, only nearly 2/3 of the patients with AN reported the same habit. The authors pointed out that a higher incidence of erosion was not observed in the patients with BN, which could be attributed to their oral hygiene practices and rinsing habits after vomiting. They explained that patients with bulimia are more likely to adopt good oral hygiene practices and seek regular dental care, which could be the reason behind the lower incidence of erosion.

### 4.3. Dental Erosion in Non-Distinguished Eating Disorders

Often, the authors of the studies cited in our review did not directly distinguish individual diagnoses of Eds, but recognised them together as EDs. Undoubtedly, AN and BN have many common features, which in the context of dental erosion seem to be quite consistent because in both diseases, patients may habitually vomit. Thus, the main factor of tooth erosion in these groups is vomiting, which acidifies the oral environment.

In the study by Garrido-Martínez et al. [35], the degree of dental erosion was significantly greater in EDs. Overall, 76.3% of the ED patients had dental erosion, while in the control group, it was only 9.2%. Additionally, the groups showed a significant difference in non-stimulated salivary flow. Among the ED participants, only 28.8% had a normal salivary flow, while the others experienced reduced flow or hyposialia. However, all of the control participants, except for one, had a normal salivary flow. The ED patients had a mean non-stimulated saliva flow of 0.23 mL/min, which was significantly lower than the normal flow rate. Approximately 20.3% of the ED patients had a non-stimulated flow of less than 0.1 mL/min, indicating hyposialia.

Women with EDs showed significantly higher dental erosions than the healthy controls. This conclusion was shared by Emodi-Perlman et al. [34], who examined women hospitalised because of chronic EDs. The ED group was divided according to their habit of daily vomiting. Dental erosion occurred in 17.6% of the non-vomiting patients and 33.3% of the vomiting patients. On the other hand, in the control group, no patients had dental erosion. Similarly, Szupiany-Janeczek et al. [59] found that individuals displaying symptoms of EDs had a higher chance of experiencing dental erosions (28.81% of cases) compared to those without symptoms (3.33% of cases). Interestingly, in this study, nearly 1/4 of the study group were males. 

In contrast, Rungta and Kudpi [54] did not find a statistically significant association between dental erosion and EDs. It can be assumed that the lack of a statistically significant association between dental erosion and EDs is the result of the fact that the study involved an adolescent female cohort. As mentioned earlier, the age of the patients and the ED duration in the case of dental erosion is not insignificant.

Most papers on EDs focus on dental erosions as a prominent oral symptom. However, Panico et al. [50] took a different approach. Surprisingly, only two patients with EDs had dental erosions, whereas the control group had none. The patients with EDs in this study may have maintained stricter oral hygiene, leading the authors to speculate that dental decalcification is not necessarily an early symptom of EDs.

In a study conducted by Hermont et al. [38], the occurrence of dental erosion was compared in cohorts of female adolescents with and without risk behaviour for EDs. The severe risk behaviour for EDs was significantly associated with tooth erosion (OR = 10.0, 95%CI: 2.5–39.4). The study also highlighted the role of socioeconomic factors in the concomitance of EDs and tooth erosion, showing that students from private schools proportionally presented more cases of risk behaviour for EDs. The link between symptoms of EDs and higher socioeconomic status is not just a referral artifact, but is evident in a representative community sample. Additionally, the authors concluded that adolescents with severe risk behaviour for EDs had a higher chance of tooth erosion when consuming citric fruit and ketchup and brushing their teeth shortly after eating.

### 4.4. Study Limitations

Undoubtedly, our systematic review with meta-analysis highlighted and summarised a significant relationship between dental erosion and EDs, considering the analyses for the specific subgroups. Among the limitations, it is certainly worth mentioning the broad timeframe of the included studies. As is well-known, the methodology and results of studies were reported differently in the past than they are now. In some previous studies, the sample size might seem relatively small. At present, EDs and dental erosion are more common among young people. The included studies also differed in the diagnostic criteria for erosive defects that have evolved over the years; however, this did not significantly affect the relationship we studied. 

Among the main sources of bias, the absence of data regarding blinding, randomisation and sample size justification should be mentioned. Moreover, it should be emphasised that, in most of the studies, the participants were women, which can be explained by the fact that EDs statistically affect women more often, and among men, they are a bit of a taboo subject. Some authors also did not separate subgroups of diagnoses of patients with EDs, which concealed the factual relationships, as it can be observed that bulimics have a greater predisposition to erosion than anorectics. Similarly, only a few studies included self-induced vomiting as a confounder, allowing a comparison of the erosion risk for vomiting and non-vomiting patients. For further studies, it would be useful to look at this relationship in a multi-factor manner, considering confounding variables, which could allow for meta-regression in the future.

## 5. Conclusions

Eating disorders are associated with an increased risk of tooth erosion. In particular, patients with bulimia nervosa and patients with self-induced vomiting had significantly higher odds of erosive lesions. Also, more than half of bulimics experienced dental erosion.

## Figures and Tables

**Table 1:** Inclusion and exclusion criteria according to the PI(E)COS.

Parameter	Inclusion Criteria	Exclusion Criteria
Population	Patients aged from 0 to 99 years, both genders	
Intervention/Exposure	eating disorders (e.g., bulimia nervosa, anorexia nervosa)	other diseases (e.g., gastrointestinal reflux)
Comparison	not applicable	
Outcomes	prevalence of dental erosion	only severity of dental erosion or only other dental indices
Study design	case-control, cohort and cross-sectional studies	literature reviews, case reports, expert opinion, letters to the editor, conference reports
	published until 27 August 2023	not published in English

**Table 2:** The characteristics of included studies.

Author	Setting	Study Group (F/M; Age)	Control Group (F/M; Age)	ED Diagnoses	Clinical Criteria for Dental Erosion
Altshuler et al., 1990 [32]	USA	40 (40/0), 23.8 ± 5.5	40 (40/0), 24.9 ± 6.1	BN	loss of enamel with exposure of dentine and/or alteration of morphology
Cavalcanti et al., 2020 [33]	Brazil	100 (100/0), mean 16.1	100 (100/0), mean 16.1	BN	based on the O’Sullivan index
Emodi-Perlman et al., 2008 [34]	Israel	79 (79/0), 23.5 ± 3.5	48 (48/0), 24.6 ± 3.0	chronic EDs: BN (*n* = 29), AN (*n* = 24), EDNOS (*n* = 16), mixed-diagnosis (*n* = 10)	4-graded scoring system (0-no, 1-enamel, 2-dentine, 3-pulp)
Garrido-Martínez et al., 2019 [35]	Spain	59 (59/0), range 19–44	120 (120/0), range 19–44	EDs: ARFID (*n* = 22), AN (*n* = 16), BN (*n* = 6), EDNOS (*n* = 15)	the degree measured according to Johansson et al. (1996)
Giraudeau et al., 2021 [36]	France	50 (48/2), mean 26.8	n/a	EDs: BN (*n* = 26), AN (*n* = 24)	BEWE scoring system using asynchronous telemedicine
Hellström, 1977 [37]	Sweden	39 (38/1), range 14–42	n/a	AN	diagnostic criteria given by Pindborg (1970) and Eccles and Jenkins (1974)
Hermont et al., 2013 [38]	Brazil	20 (20/0), range 15–18	80 (80/0), range 15–18	BN	based on the O’Sullivan index
Hurst et al., 1977 [39]	UK	17 (14/3), range 13–33	n/a	AN	different patterns: palatal, labial or generalised
Johansson et al., 2012 [40]	Sweden	54 (50/4), mean 21.5	54 (50/4), mean 21.5	EDs: AN (*n* = 14), BN (*n* = 8), EDNOS (*n* = 32)	the degree measured according to Johansson et al. (1996)
Jones and Cleaton-Jones, 1989 [41]	RSA	11 (11/0), 29.8 ± 8.4	22 (22/0), 28.9 ± 9.0	BN	4-graded scoring system (0-no, 1-enamel, 2-dentine, 3-pulp)
Jugale et al., 2014 [42]	India	50 (50/0), range 20–25	67 (67/0), range 20–25	EDs	perimolysis
Lifante-Oliva et al., 2008 [43]	Spain	17 (17/0), 20.1 ± 5.6	n/a	BN (*n* = 10), AN (*n* = 7)	4-graded scoring system (0-no, 1-enamel, 2-dentine, 3-pulp)
Manevski et al., 2020 [44]	Serbia	30 (28/2), 24.6 ± 4.4	30 (28/2), 24.7 ± 5.8	BN	BEWE scoring system
Milosevic et al., 1997 [45]	UK	33 (NR), mean 27.1	n/a	EDs: BN (*n* = 28), AN (*n* = 5)	TWI
Montecchi et al., 2003 [46]	Italy	80 (76/4), mean 15	n/a	AN	NR
Ohrn et al., 1999 [47]	Sweden	81 (79/2), median 25	52 (48/4), median 24	EDs: BN (*n* = 46), AN (*n* = 3), EDNOS (*n* = 25), mixed-diagnosis (*n* = 7)	classified according to a modification by Lussi et al. (1991) of the system proposed by Eccles (1979)
Otsu et al., 2014 [48]	Japan	71 (71/0), mean 31.1	n/a	EDs: AN (*n* = 35), BN (*n* = 27), EDNOS (*n* = 3), unclear diagnosis (*n* = 6)	diagnostic criteria given by Japanese Society for Oral Health (1985)
Pallier et al., 2019 [49]	France	70 (70/0), 32.1 ± 9.1	70 (70/0), 30.2 ± 4.7	AN (*n* = 36), BN (*n* = 34)	BEWE scoring system
Panico et al., 2018 [50]	Argentina	65 (65/0), mean 21.6	65 (65/0), mean 23.2	EDs: BN (*n* = 46), AN (*n* = 6), EDNOS (*n* = 13)	NR
Paszynska et al., 2022 [51]	Poland	117 (117/0), 14.9 ± 1.8	103 (103/0), 15.0 ± 1.8	AN	BEWE scoring system
Paszynska et al., 2015 [52]	Poland	25 (25/0), 21.2 ± 3.2	44 (44/0), 25.5 ± 4.6	BN	TWI
Roberts and Li, 1987 [53]	USA	47 (47/0), mean 25	n/a	AN (*n* = 17), BN (*n* = 30)	erosion of maxillary palatal surfaces
Rungta and Kudpi, 2019 [54]	India	21 (21/0), range 15–17	179 (179/0), range 15–17	EDs	NR
Rytömaa et al., 1998 [55]	Finland	35 (35/0), 25.3 ± 6.8	105 (105/0), 25.7 ± 7.0	BN	diagnostic criteria given by Eccles and Jenkins (1974)
Shaughnessy et al., 2008 [56]	USA	23 (23/0), 18.5 ± 2.9	n/a	AN	clinically detectable change in enamel smooth surface without evidence of dental caries
Simmons et al., 1986 [57]	USA	66 (66/0), median 26	n/a	BN	clinically observable features
Strużycka et al., 2017 [58]	Poland	29 (NR), 18	n/a	EDs	BEWE scoring system
Szupiany-Janeczek et al., 2023 [59]	Poland	59 (45/14), mean 30.6	60 (45/15), mean 30.7	EDs	NR
Touyz et al., 1993 [60]	Australia	30 (30/0), mean 19.6	15 (15/0), mean 22.1	AN (*n* = 15), BN (*n* = 15)	NR
Uhlen et al., 2014 [61]	Norway	66 (63/3), mean 27.7	n/a	EDs	VEDE system
Ximenes et al., 2010 [62]	Brazil	215 (NR) according to EAT-26, 248 (NR) according to BITE; range 12–16	n/a	EDs	NR

Legend: F, female; M, male; EDs, eating disorders; USA, the United States of America; UK, the United Kingdom; RSA, Republic of South Africa; n/a, not applicable; NR, not reported; BITE, Bulimic Investigatory Test of Edinburgh; BN, bulimia nervosa; AN, anorexia nervosa; EDNOS, eating disorders not otherwise specified; ARFID, avoidant/restrictive food intake disorder; BEWE, Basic Erosive Wear Examination; TWI, tooth wear index; VEDE, Visual Erosion Dental Examination.

## Data Availability

Data are available on request from the corresponding author.
